# SETMAR, a case of primate co-opted genes: towards new perspectives

**DOI:** 10.1186/s13100-022-00267-1

**Published:** 2022-04-08

**Authors:** Oriane Lié, Sylvaine Renault, Corinne Augé-Gouillou

**Affiliations:** 1 UMR 1253, iBrain, Université de Tours, Inserm, Tours, France; 2 iBrain, Team Neurogenomics and Neuronal physiopathology, Faculty of Medicine, 10 Bd Tonnellé, Cedex 1, 37032 Tours, France

**Keywords:** SETMAR, Network, Cognition, Neogene, Regulation, Primate

## Abstract

**Background:**

We carry out a review of the history and biological activities of one domesticated gene in higher primates, SETMAR, by discussing current controversies. Our purpose is to open a new outlook that will serve as a framework for future work about SETMAR, possibly in the field of cognition development.

**Main body:**

What is newly important about SETMAR can be summarized as follows: (1) the whole protein sequence is under strong purifying pressure; (2) its role is to strengthen existing biological functions rather than to provide new ones; (3) it displays a tissue-specific pattern of expression, at least for the alternative-splicing it undergoes.

Studies reported here demonstrate that SETMAR protein(s) may be involved in essential networks regulating replication, transcription and translation. Moreover, during embryogenesis, SETMAR appears to contribute to brain development.

**Short conclusion:**

Our review underlines for the first time that SETMAR directly interacts with genes involved in brain functions related to vocalization and vocal learning. These findings pave the way for future works regarding SETMAR and the development of cognitive abilities in higher primates.

**Supplementary Information:**

The online version contains supplementary material available at 10.1186/s13100-022-00267-1.

## Background

In humans, the most salient evolutionary feature is the development of increased cognitive capacities during the millions of years of Homo sapiens evolution. A better understanding of the basis of this evolution is a real challenge. Up to 20% of the inherited intelligence have been explained by complementary approaches such as behavioral, and also genetic, with candidate genes, genome-wide associations, linkage analyses and more recently, large scale genetic studies [1, 2].

In the meantime, domesticated transposable elements (TEs) have been shown to be efficient drivers for the rapid emergence of adaptative novelties, like increased cognitive capabilities and recent speciation [3]. The concept of « molecular domestication » was drawn by Miller and collaborators in 1997, with a handful of cases illustrating how transposable elements had provided TE-derived proteins that have been repurposed for/by their hosts [4]. Since, up to one hundred and fifty cases have been well-documented in higher eukaryotes, including humans [5, 6]. TEs have provided tens of thousands of TEs-derived DNA binding sites, serving for transcription factors and regulatory proteins involved in DNA or RNA metabolism [7], with a substantial fraction of TE-derived binding events sustaining cell type-specificity [8].

Here, we carry out a review of the history and biological activities of one of such domesticated gene, SETMAR, by discussing current controversies. We give an overview of *Hsmar1* remnants still present in the human genome, and their possible role as members of the SETMAR network. Then, we will take advantage of ChIP-seq and RNA-seq available data to establish a link between SETMAR, its network, the genes involved in central nervous system (CNS) development and its control. Our purpose is to open a new outlook that would serve as a framework for future work about SETMAR, in the field of cognition development.

### SETMAR history and biochemical activities

**Fig. 1:**
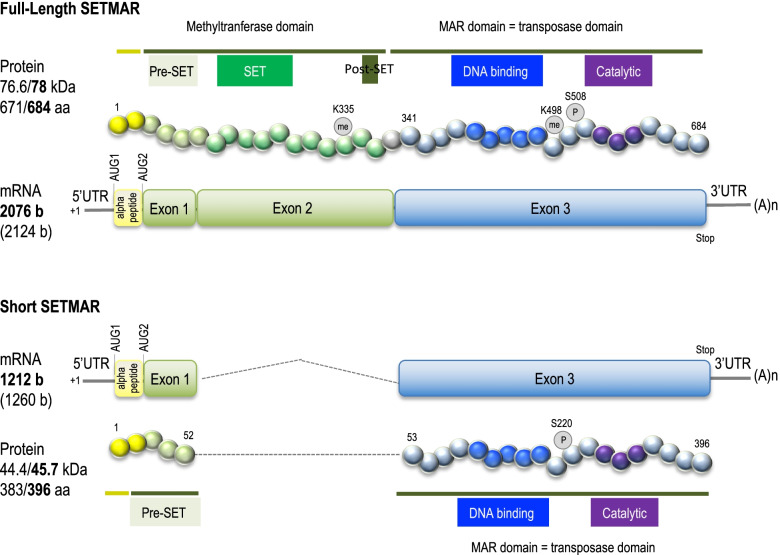
Organization of SETMAR mRNA and proteins. FL-SETMAR and S-SETMAR [12] proteins are shown. The first AUG (AUG1) leading to the translation of the alpha-peptide (in yellow) is mentioned. Known PTM are indicated. Amino-acids numbering is done for a translation starting at AUG1, with corresponding values for molecular weight and length in bold. The other values are for proteins translated from AUG2. mRNA length (in bold) are indicated according to Table 1. mRNA lengths (in brackets) correspond to the number of bases from the +1 position to the stop codon, taking into account the 33 bp 5’UTR (as sequenced in [12])

**Fig. 2:**
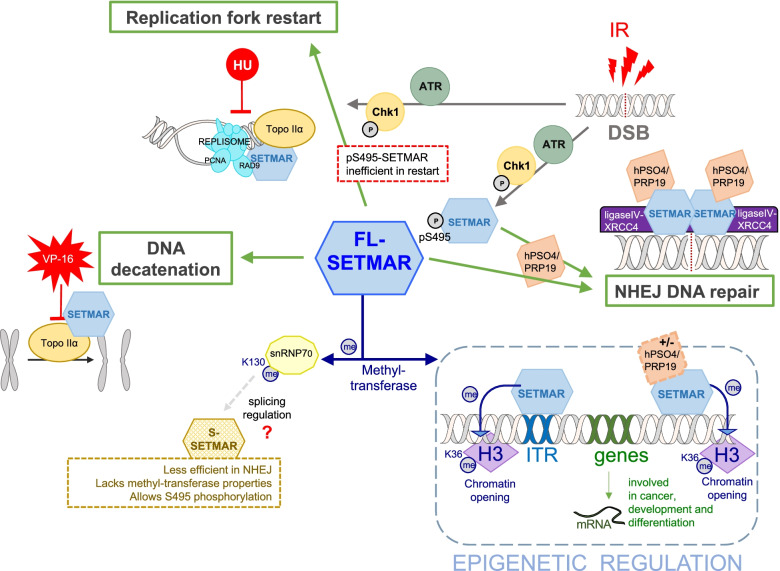
Overview of SETMAR activities, involved in replication fork restart after repair, NHEJ DNA repair, chromosomes decatenation and various epigenetics mechanisms. Usual cancer treatments are shown in red (IR: radiation, VP-16 and HU: chemicals). Cellular pathways activated or supported by SETMAR are represented in green. Identified post-translational modifications (PTM) and SETMAR partners are indicated. The picture only concerns FL-SETMAR activities because S-SETMAR activities are not well documented. So far, we know that S-SETMAR disturbs the NHEJ activity of FL-SETMAR, and does not display methyl-transferase activity

Researchers working on transposable elements of the *mariner* family (first described in Drosophila) were the first to look for such elements in the human genome and to discover a chimeric gene [9] later named *SETMAR* or *METNASE*. Born 45 million years ago, *SETMAR* is only present in higher primates [10]. *SETMAR* is made of three exons, the two first coming from the *SET* gene and coding for histone methyltransferase functions, the third coming from the *Hsmar1* transposase gene and coding for recombinase functions [11] (Fig. 1). The first activities attributed to SETMAR were logically those of the ancestral proteins now forming the neo-gene: Histone methyltransferase (for the SET domain) and DNA strand transfer (for the MAR domain) [11]. Thirties publications about SETMAR biochemical properties are summarized in Fig. 2 and described hereafter.

**Fig. 3:**
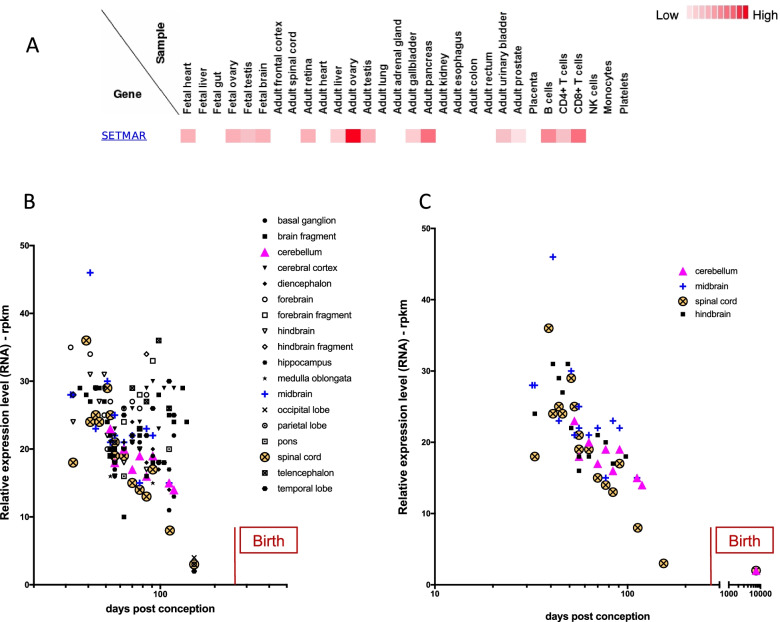
SETMAR mRNA expression in brain during human embryogenesis. **A** Data from Kim et al [13], using “SETMAR” as query. Tissues in which the SETMAR proteins were searched are indicated (tested samples). The color code is given on the right. **B** SETMAR mRNA relative level in fetal brain. 18 areas or regions are reported according to the color code. The period of birth (270 days post-conception) is indicated. **C** Details from A, with calculated Spearman correlations for cerebellum (*p*=0.027, *r*=-0.75), midbrain (*p*=0.0048, *r*=-0.72), hindbrain (*p*=0.0049, *r*=-0.74) and spinal cord (*p*=0.0003, *r*=-0.8). The period of birth (270 days post-conception) is indicated

As it will be shown thereafter, the full length SETMAR protein is described as a genome keeper, expressed in main tissues, but with different levels [11] (Fig. 3a). In cancer cells, the *SETMAR* gene is over-expressed, and the full length SETMAR sustains oncogenic processes. Under certain circumstances, *SETMAR* pre-mRNA undergoes alternative splicing, leading to the production of shorter proteins, enriched in cancer stem cells [12, 14]. Relationships between SETMAR and cell state has been since documented [14–16]. Works presented below concern the full-length protein (78kDa) that is referred as SETMAR, unless specified.

#### SETMAR in DNA binding, repair and foreign DNA integration

Like the *Hsmar1* transposase from which it comes, the MAR domain is made of a DNA binding domain and a recombinase domain. The ability of SETMAR to bind the *Hsmar1* extremities, called TIRs (for Terminal Inverted Repeats) was first investigated. As expected, SETMAR is able to interact with the 30-bp TIRs sequence, similarly to the reconstructed ancestral transposase (HSMAR-RA) [10, 17–19]. Several studies demonstrated that SETMAR specifically interacts with *Hsmar1* TIRs, whereas it does not recognize other sequences with the same efficiency. This characteristic of SETMAR is of outmost importance as thousands of TIRs are present in the current human genome, and represent as many specific sites of fixation [10]. Beck and collaborators [20, 21] also demonstrate that hPSO4/PRPF19 is a SETMAR partner that modifies its binding properties, and improves recognition and binding to sequences other than *Hsmar1* TIRs.

A second characteristic related to the SETMAR recombinase domain is its ability to integrate foreign DNA, using the DNA phosphatidyl-transferase activity shared by transposases to perform the last step of transposition, *i.e*. integration. Lee *et al* [11] measured the ability of a linearized vector to have its free ends joined by SETMAR *in vitro*. Their results suggested for the first time that SETMAR might constitute a component of the non-homologous end-joining (NHEJ) repair system. Since then, several studies have confirmed that SETMAR is an efficient partner of the NHEJ pathway, helping to produce undamaged 3’ ends suitable for patching and ligation, bypassing and removing blocked termini [22–24]. It was shown [25, 26] that SETMAR is localized at double strand breaks, where it enhances repair efficiency, and promotes the resolution of phosphorylated H2Ax foci, a marker of DNA double-strand breaks at collapsed forks. In addition, various NHEJ factors were demonstrated to directly interact with SETMAR, such as hPSO4/PRPF19 [21] and the ligase IV-XRCC4 complex [27]. Although these activities tend to be spontaneously associated to the MAR domain, a contribution of the SET domain is formally sustained both by mutations that result in a decrease in NHEJ efficiency, especially for precise repair [11] and by the use of the SET domain alone [28]. These later and recent works suggest that both the SET and the MAR domain can play a role in DNA repair and illegitimate integration when they are not linked together (with a need to clarify their exact contribution) however the whole SETMAR protein is only faintly responsible for such activities. Although inconsistent with previously mentioned studies, these results illustrate well that many unanticipated parameters affect SETMAR activity in cells. They also show that the use of an overexpressed recombinant protein is a simplifying approach/vision, which struggles to resolve complex issues.

#### Cell cycle and replication

Upstream of phosphatidyl-transferase activity, transposases need to perform DNA double strand breaks to excise the transposon at the beginning of transposition. These properties (concerted cleavage and re-ligation) are both the main features of chromosomes decatenation, the process whereby sister chromatids synthetized during the phase S are untangled to ensure proper chromatid segregation in mitosis, to prevent chromosomes break during anaphase and inappropriate repair [29]. Accordingly, it was demonstrated that SETMAR physically interacts and co-localizes with Topoisomerase IIα (Topo IIα), the key chromosome-decatenating enzyme [30, 31]. Furthermore, it enhances Topo IIα decatenation and increases cell resistance to Topo IIα inhibitors [32], commonly used as chemotherapeutic drugs. Topo IIα is also required during replication, to unwind the DNA before the replication fork, allowing the fork to progress during DNA synthesis. Cells are particularly vulnerable to DNA damage during replication as multiple lesions can cause replication forks pauses, even to stall, until DNA breaks are repaired. The replication fork restart to complete replication is an important process of cell cycle. Information about the role of SETMAR in the replication stress response, through helping replication forks restart, was addressed by hydroxyurea (HU) treatment, a chemical that blocks replication. It was demonstrated that SETMAR knock-down improves sensitivity of the cells to the HU treatment, delaying the response and the restart of DNA synthesis [26]. The involvement of SETMAR during the phase S was confirmed since it co-immunoprecipitates with PCNA and RAD9, members of the PCNA-like RAD9-HUS1-RAD1 intra-S checkpoint complex [26]. Taken together, these works sustain a strong involvement of SETMAR in DNA repair by NHEJ, chromosome decatenation and the replication stress response.

Because the cell cycle is regulated by a cascade of phosphorylation events initiated upon DNA damage sensing, and because transposases of the *mariner* family were proven to be regulated by phosphorylation [33], phosphorylation events between SETMAR and CHK1 were investigated. It has been shown that DNA damage by ionizing radiation induces SETMAR phosphorylation on Ser-495 (pS495) by CHK1 [34]. pS495-SETMAR is inefficient in the restart of collapsed replication forks, but it is recruited to DNA double strand breaks more efficiently to enhance repair. Thus, SETMAR is one of CHK1 effectors in replication stress, to protect the cell and repair the DNA breaks. In addition, pS495-SETMAR increases CHK1 stability [35]. A recent study proposes that SETMAR could be present at replication origins, but this point remains to be confirmed by the used of synchronized cultured-cells [14].

#### What about the SET domain?

Histone methyltransferase activity of SETMAR was also one of the first investigated in pioneer works. Using *in vitro* approaches, Lee *et al* [11] have demonstrated that SETMAR is able to di-methylate H3K4 and H3K36, two epigenetic markers of open and accessible chromatin. Although this data have been since challenged [36], SETMAR dimethylation of H3 at K36 was recently confirmed by an overall epigenetic approach comparing overexpressed recombinant SETMAR mutated or not in the SET domain [37]. The *in vivo* ability of SETMAR to methylate H3K36me2 was also confirmed by the knockdown of SETMAR expression that dramatically decreased H3K36me2 level in glioblastoma cell lines [24]. The ability of SETMAR to promote H3K36me2 is relevant beyond its role in NHEJ, by enhancing the opening of chromatin loci that need to be repaired. Xie *et al* [15] also mentioned a possible involvement of SETMAR in H3K27me3 marks which are detected in bladder cancer cells. It seems important to note that these findings concern an atypical *SETMAR* transcript (variant 5, see Table 1) instead of the one coding the FL-SETMAR. Variant 5 is coding for a protein that was never detected *in vivo* (SETMAR-L) and that does not contain a full SET domain. Even if translated, *SETMAR-L* mRNA would allow the production of a SETMAR protein close to S-SETMAR, that cannot directly explain the H3K27me3 marks which are detected in bladder cancer cells [15].

**Table 1:**
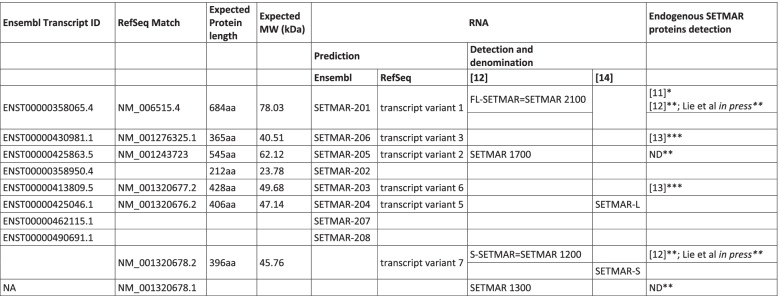
Annotation of SETMAR variants found in literature and databases

In addition to histone H3 methylation, SETMAR was demonstrated to promote both auto-methylation (at K335) and methylation of the splicing factor snRNP70 at K130 [36]. In these cases, SETMAR is rather a mono-methyltransferase. The proposition of Carlson *et al* [36] about SETMAR auto-methylation at K335 is that it concerns only a small part of the cellular SETMAR *in vivo*, and it probably does not change the catalytic activities of the enzyme, rather the interactions with its partner-proteins. The fact that the amount of K130-me1 snRNP70 is increased in cells overexpressing SETMAR argues in favor of an involvement of SETMAR in splicing regulation, but this point remains to be explored. Finally, SETMAR has also been shown to auto-methylate on its residue K485, which regulate chromosome decatenation [31].

During the past decade, the role of the SET domain may have seemed controversial. Nevertheless, recent works, based on high-throughput analysis techniques, convince us that the SET domain is really involved in the methylation of different target proteins: histone H3 (K36), snRNP70 and SETMAR itself. Finally, it has been shown that colorectal cells do not produce SETMAR variants that contain the SET domain [14]. This observation reminds us of the importance of Western blot approaches (to detect endogenous proteins), as an essential complement to validate RNA approaches.

#### SETMAR expression

Databases (Ensembl and NCBI Gene) describe various isoforms of SETMAR transcripts. Some of them were found in different cell types [12, 38] while others were never detected *in vivo* (Table 1).

Based on databases information, five of these transcripts could encode proteins. Two publications actually show experiments detecting endogenous SETMAR proteins [12, 14]. Only one commercial antibody is indeed specific. It is directed against the N-terminal part of the protein and thus allows the detection of all putative SETMAR proteins (at least those predicted by databanks). Currently, four endogenous SETMAR proteins have been detected (Table 1): one of respective apparent molecular mass around 77 kDa [12] and three of respective apparent molecular mass around 48-52 kDa [12, 14]. Note that it seems difficult to exclude, on the basis of Western blots alone, that S-SETMAR, the short variant first described by Dussaussois *et al* [12] is not one of the two short variants recently described by Antoine-Lorquin *et al* [14].

#### SETMAR translation

Until 2014, the methionine commonly used as the first SETMAR amino acid (aa) was located at ATG2 (Fig. 1). Jeyaratnam *et al* [38] were the first to propose that the SETMAR open reading frame could start at ATG1, leading to a protein of 684 amino acids, *i.e*. including the 13 aa sequence that was later called “peptide alpha” [14]. All experiments using recombinant SETMAR were performed with a full length SETMAR variant of 671 aa, without “peptide alpha” (see Fig. 1). This led to two different numbering systems of SETMAR amino acids. For instance, the phosphorylation of the MAR domain is described in the literature at position S495 [35], *i.e*. in the 671 full length SETMAR, whereas the methylation of the SET domain is described at position K335 [36], *i.e*. in the 684 full length SETMAR. We now propose that the community should adopt the numbering which takes into account the protein translated from ATG1, as it is done in Fig. 1.

### *Hsmar1* remnants and the associated SETMAR network

Transposases are the most abundant and ubiquitous genes in nature [39]. They are at the origin of hundreds of “neo-genes” created by the domestication of various TEs during evolution [40], as was *Hsmar1*. The first *Hsmar1* copy was inserted in an ancestral primate genome nearly 45 million years ago [10]. After millions of years of transposition and amplification, hundred copies of *Hsmar1* were created. This burst of amplification was followed by *Hsmar1* inactivation, due to accumulated mutations and deletions within both the transposase gene and/or TIRs sequences. Today, the human genome contains about 250 defective *Hsmar1* copies and nearly 12,500 TIRs organized either in solo TIR or miniature elements lacking the transposase coding gene and called MITEs [41], both coming from either partial excision or recombination for full-length elements.

#### hsa-miR-548

Because MITEs display a nearly perfect palindromic structure, with TIRs separated by a few base pairs in hairpin structures, it has been proposed that they can be on the origin of short endogenous small non-coding RNAs, siRNA and/or miRNA, that play a major role in gene regulation at the post-transcriptional level. In 2007, *Hsmar1* derived MITEs were demonstrated to be at the origin of an active miRNA called hsa-miR-548 [42]. Since, more than 75 hsa-miR-548 genes were found in the human genome, leading to the expression of about 80 miRNA targeting an estimated 1000 to 6000 genes, depending on the database used (miRBase, miRWalk, miRanda and TargetScan). Over 80 papers have been published demonstrating the implication of hsa-miR-548 in the regulation of genes involved in diverse pathologies such as cancers (breast, bladder, liver, … ), ischemia or coronary artery disease [43–47]. In addition, SETMAR mRNA are predicted to be targets for (at least) 11 hsa-miR-548 (miRWalk database).

#### Hsmar1 TIRs

Among the nearly 12,500 *Hsmar1* TIRs still present in the modern human genome, about 4,000 display a sequence allowing SETMAR binding [41, 48], promoting the organization of what is referred herein as the “SETMAR network”. The genetic control potentially associated to this network can occur in various ways: (1) via the SET domain of SETMAR, (2) by competing or interacting with partner factors, or (3) via regulatory mechanisms repressing TEs activity. This last hypothesis has been verified in both human and non-primate cells (*i.e*. HeLa *versus* CHO cells), in which an active (reconstructed) *Hsmar1* copy was stably inserted within a construction allowing the tracking of the recombinant *Hsmar1* excision. Interestingly, the genetic background of the naive CHO cells is permissive for *Hsmar1* transposition, whereas HeLa cells are not [41]. The authors demonstrate that the recombinant *Hsmar1* copy is silenced both by DNA methylation and H3K9me3 depositions. In contrast, endogenous *Hsmar1*, TIRs or SETMAR are not specifically silenced. They display similar epigenetic characteristics than the whole human genome. This overall approach does not rule out the possibility that some *Hsmar1* sequences could be the target of epigenetic regulations, possibly cell-specific, but this remains to be analyzed in details. The fact that various *Hsmar1* TIRs remain accessible strengthens the idea that SETMAR represents a good candidate to implement a (epi)-genetic network. Tellier and Chalmers have recently investigated this hypothesis. They show that SETMAR overexpression induces a shift of mRNA expression by more than 2-fold of almost 1,500 genes in cultured osteosarcoma cells (U2OS) [37]. The shift of expression is highly correlated to the SET domain activity. Using a ChIP-seq approach, they demonstrate that genes with a TIR bound by SETMAR (157 genes) are strongly over-represented amongst the 374 immunoprecipitated genes. Nevertheless, 47 immunoprecipitated genes contain a TIR that is not occupied by SETMAR. In 227 genes, SETMAR recognizes and binds sequences other than *Hsmar1* TIR. Although it has recently been contradicted in a quite artificial model (haploid cell line with KO of endogenous *SETMAR* gene and overexpression of a recombinant one) [48], this observation strongly supports the results of Beck and collaborators [21], showing that hPSO4/PRPF19 is a SETMAR partner that modifies its binding properties (see below). The SETMAR complexes containing hPSO4/PRPF19 display less specific binding properties, expanding the field of SETMAR network and the number of possible target genes, as illustrated in two recent works [14, 37]. Finally, genes controlled by SETMAR in Tellier and Chalmers study [37] are described as being involved in organ development and/or differentiation cellular events that are mis-regulated in cancer cells. Surprisingly, the genes on which SETMAR binds with a significant change in expression rate (2-fold) shows an unexpected enrichment of genes involved in synaptogenesis or certain cognitive skills such as language learning. So, 12 years after the hypothesis of a network associated with SETMAR has been proposed [10], it is experimentally confirmed. Future works will have to deal with peculiar challenging issues, as the identification of truly used SETMAR binding sites or the biological consequences of SETMAR binding on target genes. Another challenge concerns the role and occurrence of SETMAR variants. Indeed, Antoine-Lorquin *et al* [14] have shown that colorectal cells (cancerous or not) do not express the full-length form of SETMAR (see below), imposing a hypothesis that had been barely suggested by Dussaussois et al [12]: SETMAR alternative splicing could be tissue or cell-specific. This new hypothesis should lead us to reconsider what we had taken for granted: SETMAR is probably not a ubiquitous protein.

### SETMAR and diseases

#### SETMAR and cancer

Some studies suggest that SETMAR amounts positively correlates with cell proliferation [26, 30, 36], whereas another does not suggest it [28]. If cell proliferation correlates positively with SETMAR, then cancer cells are expected to over-express it. Accordingly, SETMAR protein has been searched in tumor from patients, such as hematologic neoplasms [38], gliomas and glioblastomas [12]. In those tissues, SETMAR mRNA are increased up to 70 times (depending on the sample analyzed) when compared to healthy tissues. SETMAR mRNA are the result of various alternative splicing. Among them, the mRNA coding the complete SETMAR protein (FL-SETMAR, for full-length SETMAR) is always the most abundant. Ten different mRNA have been described in hematologic neoplasms (acute myeloid leukaemia (AML), mantle cells lymphoma and chronic myeloid leukaemia), and four in gliomas and glioblastomas, which are identical to four of those found in the hematologic neoplasms. According to their sequences, three of the six mRNA encode functional proteins: the full-length protein and for proteins lacking either a part of the SET domain, or the whole SET domain and a part of the pre-SET domain. Yet, they all contain the whole MAR domain [12, 38]. In AML, the increased expression of the full-length mRNA was correlated with a low level of chromosomal translocation, suggesting a protective effect of high SETMAR expression [38]. Until recently, the presence of SETMAR proteins in tumor tissues from patients was addressed only in glioblastomas (GBM) [12]. Two variants have been detected, the full-length SETMAR and a truncated form (S-SETMAR for short SETMAR) lacking a part of the pre-SET domain and the SET domain, both encoded by exon 2. The mRNA encoding this short variant has been previously detected in hematologic neoplasms. Quite similar proteins have been also recently detected in colorectal cancers [14]. In GBM, the relative abundance of each SETMAR variant does not match the relative abundance of the related mRNA, since both SETMAR proteins are enriched in GBM, whereas only the full-length mRNA is over-represented. Dussaussois et al [12] have demonstrated that S-SETMAR is still able to perform NHEJ but with less efficiency than FL-SETMAR, thus confirming the importance of the SET domain in SETMAR functions. In order to understand the intriguing fact that S-SETMAR is over-represented (related to its mRNA level), the role of a short N-terminus sequence of 13 amino acids was addressed, and evidences were provided that this peptide (called alpha-peptide) increases the half-life of both SETMAR proteins from hours to days [12]. At that time, the lack of anti-alpha-peptide antibodies did not make possible to check the presence of this peptide in endogenous SETMAR. However, very recent data confirm that most endogenous SETMAR contain an alpha-peptide [49]. The role of each SETMAR protein has to be defined, but a first line of evidences suggests that S-SETMAR is preferably expressed in cancer stem cells, whereas FL-SETMAR is preferentially expressed in differentiated cells. *SETMAR* was demonstrated to be involved in the recurrence of GBM, since its expression is upregulated in therapy resistant cells [24]. The resulted high level of H3K36me2 contributes to activate the NHEJ repair pathway. Expectedly, the treatment of therapy resistant cells with sh-*SETMAR* reduces the overall level of H3K36me2 and leads to the irreversible senescence of the cells [24]. Data recovered from GBM and GBM stem cells are reinforced by colon cancer stem cells data [16]. Here, the knockdown of SETMAR by siRNA against exon 3 (coding the domain MAR, present in all SETMAR proteins, see Fig. 1) is directly related to the down-regulation of stemness factors as NANOG, OCT3/4 and SOX2. Even if these findings do not allow discriminating between S- and FL-SETMAR, they clearly establish a relationship between SETMAR and stemness and/or differentiation. This has been supported by recent findings demonstrating that NONO (a factor that plays a role in gene activation and mRNA processing during cell differentiation [50]) is needed to perform SETMAR exon 2 retention in bladder cancer cells [15]. This reinforces the hypothesis linking SETMAR isoforms and cell differentiation. The only weak point of Xie et al findings [15] is that their works concern an atypical *SETMAR* transcript (variant 5, see Table 1) instead of the one coding the FL-SETMAR.

According to these results, a first working hypothesis can be proposed. The FL-SETMAR could be a genome keeper, expressed (at the mRNA level) in almost all adult healthy tissues [11], but with variable amount at the protein level [14]. Its role may be to improve the efficacy of biological mechanisms otherwise present in species other than higher primates. In cancer cells, the *SETMAR* gene is over-expressed, and FL-SETMAR then sustains oncogenic processes and the development of cancer cells or cancer stem cells. Under certain circumstances, SETMAR pre-mRNA undergoes alternative splicing, resulting in shorter SETMAR isoforms, containing or not the alpha-peptide. It cannot be excluded that alternative splicing occurs differently depending on the cell type, leading to the production of different short variants following tissue-specificity. These shorter and possibly more stable proteins could disrupt the activity of the FL-SETMAR (by competing with partner-proteins, with DNA binding sites, by heterodimers assembly between S- and FL-SETMAR … ). The fact that high level of S-SETMAR (the short variant found in GBM) is a factor of good prognosis for patients with GBM [49] could be a consequence of this dominant-negative activity.

#### SETMAR as a therapeutic target

As previously mentioned, SETMAR is seen as being a putative obstacle to the classical treatments of cancer involving radiotherapy and chemotherapy (Fig. 2). It thus appeared interesting to develop SETMAR inhibitors to improve cancer treatments. Various SETMAR inhibitors have been screened on the basis of the MAR domain strand-transfer activity [51]. Of particular interest, ciprofloxacin impairs the SETMAR ability to cleave and to repair DNA. This increases the sensitivity of cancer cells and xenograft tumor models to already clinically available chemotherapy, by blocking the repair of chemotherapy-induced DNA damage. On the other hand, several hsa-miR-548 have been described to be directly involved in oncogenesis; for instance, miR-548a-3p repressed SIX1, a transcription factor that controls glycolytic genes. In breast cancer, SIX1 is over-expressed following the decrease of miR-548a-3p level, and this level is a good predictor of prognosis in patients [52]. Another hsa-miR-548, miR-548b, was shown to inhibit proliferation of glioma cells (from brain tumors) by repressing MTA2, the metastasis tumor-associated protein-2 [53]. In both cases, therapeutic approaches can be considered by the restoration of an increased level of hsa-miR-548.

#### SETMAR and integrative viruses

Because SETMAR was identified as being important for the integration of foreign DNA into the host cell genome, Williamson et al [54] examined whether SETMAR expression levels could have an influence on lentiviral genomic integration (more precisely, the VSV-G pseudo-typed HIV1-backbone lentivirus). They show that the expression level of SETMAR correlates with live lentiviral integration and that this activity relies on the MAR domain efficiency. In contrast, SETMAR has no effect on the amount of either total cellular viral RNA, cDNA or 2-LTR circles.

#### Other diseases?

In order to identify a potential role of SETMAR in genetic hereditary diseases, two complementary approaches have been used.

First, large genetic alterations involving *SETMAR* have been collected using ClinVar data (Table 2). No case of deletion or duplication of the *SETMAR* gene alone has been reported. In nine cases, *SETMAR* and *SUMF1* (only) are co-deleted, but no clinical significance is related. This codeletion is expected since SETMAR belongs to a SUMF1 intron.

**Table 2:** Summary of ClinVar data involving *SETMAR*

Alterations	Nb of involved genes	n	Clinical significance
Deletions	> 20	50	pathogenic
	4 to 20	21	pathogenic to likely pathogenic
	< 4	15	from likely benign to uncertain significance
	SETMAR alone	none	
duplications	> 20	19	From pathogenic to uncertain significance
	4 to 20	4	from pathogenic to uncertain significance
	< 4	3	uncertain significance
	SETMAR alone	none	

Genetic alterations are either deletions or insertions and concerned a variable number of genes (over 20 to SETMAR alone). The number of observed cases is given (n), as well as the clinical relevance

Second, punctual or little mutations within the *SETMAR* gene have been examined, using the Genome Aggregation Database (gnomAD) of the Broad Institute. Synonymous and missense SETMAR loss-of-function protein variants are scored for their expected *versus* observed occurrences, giving a ratio of about 1 (0.98 and 0.96 respectively), suggesting that the *SETMAR* gene is under low (or no) selection. This is confirmed by a pLI (probability of being loss-of-function intolerant) of 0, meaning that SETMAR could tolerate mutations without displaying disease phenotypes. Indeed, many individuals in the general populations display heterozygous deletions of this part of their genome (Database of Genomic Variants), with a heterozygous status implying that deletions or punctual mutations are not deleterious. In contrast, Cordaux *et al* [10] have shown that the N-terminal half of the MAR domain (*i.e.* the DNA binding domain) is under strong purifying selection, and this was recently confirmed by Tellier *et al* [37]. This point has recently been completed by Antoine-Lorquin *et al* [14], who consider that the whole SETMAR sequence is under strong purifying selection. This apparent contradiction can be solved if we consider that the SETMAR variants identified in gnomAD exist only as heterozygotes. It cannot be ruled out that homozygous mutations would have a phenotypic impact.

In conclusion, based on the gnomAD and pLI, SETMAR does not seem to be an essential gene, *i.e* can tolerate mutations without displaying disease phenotypes, at least as heterozygous, but rather to be implicated or associated to cancers with changes in expression levels associated to alternative splicing. A new hypothesis should be considered for future studies: SETMAR as a gene, *i.e.* including all the transcripts, can be considered ubiquitously expressed while it displays tissue- and cell-type specificities upon regarding each of the transcripts.

### SETMAR and cognition

In a recent review, Roth and Dicke [2] investigate whether the higher cognitive abilities, characteristic of human are specific or shared by non-human animals, even in preliminary forms. They check about nine behavioral items or abilities (tool use or fabrication, problem solving, gaze following, mirror self-recognition, imitation, metacognition, theory of mind, conscious attention, prosocial behavior). Their results have shown that none of these abilities can be regarded as unique to humans. They all have precursors in non-human primates. More, young children (before the age of three) abilities do not really differ from those of apes and old-world monkeys. Good correlations are observed between information processing capacities and the number of cortical neurons, their packing density and axonal velocity. For primates, the human brain constitutes an optimization of these characteristics, relying on cortex development and its reorganization during evolution. Implemented for solving problems arising from social, ecological, practical and mental fields, these increased information processing allowed the most remarkable difference between human and non-human animals, *i.e.* the occurrence of articulated, syntactical and grammatical language [2].

The above makes it possible to distinguish three stages in the evolution of primates’ mental abilities, which coincide with the phylogenetic classification usually accepted for these primates: the first one is represented by prosimians and new world monkeys, the second by old-world monkeys and great apes, together with human young children and the third by older humans. Because *SETMAR* is concomitant to the anthropoid lineage (old-world monkeys, great apes, humans), it is tempting to consider whether it could be one of the actors of cortex development and higher brain organization.

In 2018, Florio and collaborators published a cutting edge study [55] which proposed to identify genes involved in the expansion of the human’s neocortex by searching those early and specifically expressed in progenitors of fetal neocortex (cCNP), from five distinct transcriptomes. They identified 35 primate-specific genes (which do not have orthologs in non-primate genomes), completed by 15 human-specific genes, all 50 preferentially expressed in cCNPs. Among them, 17 genes are shared by primates of the anthropoid lineage. All have evolved from the duplication (partial or complete) or retroposition of ancestral genes. 10 of them are coding DNA binding proteins of the zing finger (ZNF) family, the other 7 being involved in various cellular processes (tRNA maturation, immunity, lipids transport and metal ions homeostasis). Together, they represent a first set of candidate genes for the expansion of the anthropoid primates’ cortex. Another set of candidate genes can been provided by the genes co-opted by anthropoid primates from TEs [56]. Among them, SETMAR has characteristics that make it a fascinating candidate which could have contributed to the development of higher primates’ cognition, including human. Although it has never been considered in this light, we propose here an original hypothesis regarding the evolutionary functions of SETMAR. This hypothesis takes into account some of biological and biochemical properties of SETMAR, its expression profile (at protein and mRNA levels), the high selection pressure to which it is subjected, its link with a network of transposon-derivate DNA binding sites, and gene ontology of SETMAR bound genes. SETMAR exact biological functions are not yet well know, and some aspects are still under debate. Other SETMAR properties have never been completely explained, such as its increased expression in various cancers and its related network, constituted by *Hsmar1* binding sites.

#### SETMAR expression during human embryogenesis

Mass spectrometry data from LC-MS analysis performed by Kim et al [13] have shown that in the human fetus, the SETMAR protein is moderately detected in the whole brain, the gonads and the heart. Interestingly, the expression profile in adult is radically different, the SETMAR protein being detected in B cells, the pancreas, the bladder, the gallbladder, gonads and the retina, but not in the whole brain (Fig. 3a). These data do not allow to distinguish between SETMAR variants since the peptide detected for analyses (AIFLFEFK) comes from the beginning of exon 3. This exon is coding for the MAR domain, the domain shared by all SETMAR proteins.

More accurate analyses have been done at the mRNA level, within various specific zones of the brain during early embryogenesis. Data from Ensembl web site were collected from projects concerning the fetal brain and analyzed (see Supp Data for details). They have obviously shown that the overall level of SETMAR mRNA expression decreases upon embryogenesis, reaching a faint level at the end of pregnancy (Fig. 3b). For four areas (Cerebellum, midbrain, hindbrain and spinal cord), the decrease of SETMAR mRNA expression is statistically correlated with time, as verified with Spearman test (Fig. 3c). Although such correlations alone are not sufficient to make or support our hypothesis, nevertheless they allow to consider a role for SETMAR during neurogenesis.

#### SETMAR networks in brain

RNA expression data from BrainSpan database have been used to identify SETMAR co-expressed genes during human brain development (0-38 mpc), assuming that gene co-expression networks capture biologically important patterns [57] (see Supp Data for details). We thus looked for (i) genes that are co-regulated with SETMAR upon brain embryogenesis and (ii) within these genes the ones that could be SETMAR target genes because they contain *Hsmar1* TIR in their sequences. The 500 most strongly co-regulated genes (positively [r between 0.648 to 0.993] or negatively [r between -0.557 to -0.834]) were retained for analysis (list#1 and list#2, Table S1). Gene ontology [58] shows that most of the positively co-regulated genes are involved in translation and transcription regulations, whereas the negatively co-regulated genes are involved in cations transmembrane transport (Table S2). These findings match well the gene ontology displays for Florio et al genes list [55] (ZNF proteins, tRNA maturation, lipid transport and metal ions homeostasis). These initial findings are far from sufficient to conclude that SETMAR plays a role in neurogenesis, let alone in the cognitive abilities of higher primates. We thus pursue our analyses by comparing several set of genes: genes containing at least one TIR [41] (list#3), genes from Florio et al studies [55] (list#5), SETMAR positively and negatively co-regulated genes (list#1 and #2), and genes involved in intellectual disability (ID) (list#4), available at the IDGenetics website. The complete genes list is given in Table S1. Our main findings are presented in Tables 3 and 4.

**Table 3:** Gene sets used in our analyses

	SETMAR positive co-regulated genes during brain dev.	SETMAR negative co-regulated genes during brain dev.	Florio *et al* genes	ID involved genes
Number of genes	500	500	50	816
Genes with SETMAR TIR	11	17	2	60

Genes with SETMAR TIR were identified using the InteractiVenn web site [59]

**Table 4:** Common genes between sets defined above

	SETMAR (+) co-regulated genes during brain dev.	SETMAR (-) co-regulated genes during brain dev.	Florio *et al* genes	ID involved genes
SETMAR (+) co-regulated genes		0	0	**16 (2)**
SETMAR (-) co-regulated genes	0		**7**	**19 (1)**
Florio *et al* genes	0	17		0

Common genes were identified using the InteractiVenn web site [59]. Values in bracket show the number of genes with a SETMAR TIR among the total number of genes. The complete lists of genes are given in Table S1

Three genes, involved both in ID and co-regulated within SETMAR upon brain development, display a SETMAR binding site in their sequences (LRPPRC and PDE4D (positively co-regulated); CYP27A1 (negatively co-regulated)). Overall, the gene ontology of the different groups does not give anything more or better than the previously mentioned categories. The remarkable exception comes from the 60 genes involved in ID and having a SETMAR binding site (TIR), for which gene ontology shows highly significant enrichments for genes involved in vocal learning (fold enrichment > 100) and in vocalization behavior (fold enrichment = 51.5). These genes are NRXN1, CNTNAP2 and GLI3. Vocal learning and vocalization are essential features of cognition development. These findings therefore support our hypothesis, that remains to be experimentally confirmed. Indeed, results presented here are based solely on correlations between data from different databases.

#### Other SETMAR networks

Tellier and Chalmers [37] have used ChIP-seq analyses to describe for the first time genes bound by SETMAR in human cells. Albeit this work has not be done with cells from CNS, it is the only one describing biological interaction between SETMAR and target genes. 279 genes have been found to be physically targeted by SETMAR (List #6, Table S1). Among them, 28 are up-regulated more than two fold (list#7, Table S1). The gene ontology shows that among the eight most represented categories, seven concern brain specific biological processes ([37] see supplemental data): synapse assembly and functions (5 items), learning and memory (1 item), vocalization (1 item). Moreover, the most enriched genes are those involved in vocalization behavior (fold enrichment >100), as found within the 60 genes involved in ID that have a SETMAR binding site in their sequence. Taken together, these analyses display two short lists of genes involved in the same pathway, *i.e*. vocal learning, for which SETMAR may be an important regulator. Tellier and Chalmers [37] identified genes are NRXN1, NRXN3 and SHAK2, and the genes we identify are CNTNAP2, GLI3 and NRXN1.

These findings are very surprising, because gained through different approaches, but they are very challenging. They strongly support our hypothesis of an involvement of SETMAR network in cognition skills development in higher primates, and identify NRXN1 as an important piece of this network.

## Conclusion

The last review on SETMAR functions dates from 2010 [60] and since, many studies have highlighted its importance in certain cancers and its network function. Here, we give an updated review of its known functions, while bringing a cutting-edge vision of putative roles of this pleiotropic protein. In writing this review, we noticed that an important element was missing in understanding SETMAR activities. SETMAR is known to interact with DNA as a dimer [10]. What has never been explored, since the short variants were discovered, is the possibility of forming heterodimers between the different variants. If this hypothesis is correct, it opens up increased possibilities for regulation, through the possible combination of all variants with each other.

What’s newly important about SETMAR can be summarized as follows: (1) the whole protein sequence is under strong purifying pressure; (2) its role is to strengthen existing biological functions rather than to provide new ones; (3) it displays a tissue-specific pattern of expression, at least for the alternative-splicing it undergoes.

At the overall level, studies reported here demonstrate that SETMAR protein(s) may be involved in essential networks regulating replication, transcription and translation. During embryogenesis, SETMAR appears to contribute to brain development. Interestingly, SETMAR has been described to interact with genes involved in vocalization and vocal learning. Vocalizations are essential in many vertebrates, including humans, since they influence mother-infant attachment, social and behavioral development. In human, vocalization and babble are prerequisites for sounds and words learning, in other words for spoken language learning [61]. These findings pave the way for future works regarding SETMAR and the development of cognitive abilities in higher primates.

## Supplementary Information

**Additional file 1: Table S1.** List of genes mentioned in the main text.

**Additional file 2: Table S2.** Gene ontology of *SETMAR* and brain co-regulated genes during embryogenesis.

**Additional file 3: Supp Data.** Mat & Met for SETMAR and cognition.

## Data Availability

The datasets analyzed during the current study are available in the following repositories: • ClinVar data (https://www.ncbi.nlm.nih.gov/clinvar) • Genome Aggregation Database (gnomAD) of the Broad Institute (https://gnomad.broadinstitute.org) • Database of Genomic Variants (http://dgv.tcag.ca/dgv/app/home) • Data from Kim et al [13] http://www.humanproteomemap.org/query.php • Mass spectrometry data performed by Kim et al [13] are available on the Expression Atlas web site (https://www.ebi.ac.uk/gxa/experiments/E-PROT-1/Results?geneQuery=%5B%7B%22value%22%3A%22ENSG00000170364%22%7D%5D&filterFactors=%7B%22DEVELOPMENTAL_STAGE%22%3A%5B%22fetus%22%5D%7D) • Ensembl web site (projects about the fetal brain) (http://www.ensembl.org/Homo_sapiens/Gene/ExpressionAtlas?db=core;g=ENSG00000170364;r=3:4303304-4317567) • Transcriptomic data from the BrainSpan web site. 0-38 pcw in the human whole brain. (https://www.brainspan.org/rnaseq/searches?domain=10173,10185,10278,10194,10163,10291,10208,10209,10225,10236,10235,10243,10252,10268,10269,10294,10361,10551,10550,10552,10333,10391,10398,10665,10656,10657&stages=IIA,IIB,IIIA,IIIB,IV,V&search_type=correlation&search_term=&seed=1099763) • IDGenetics web site (Known SNVs and InDels list). http://www.ccgenomics.cn/IDGenetics/index.php

## Abbreviations

AML: Acute myeloid leukemia
cCNP: Progenitors of fetal neocortex
CNS: Central nervous system
GBM: Glioblastoma
ID: Intellectual disability
KEGG: Kyoto Encyclopedia of Genes and Genomes
LC-MS: Liquid chromatography – mass spectrometry
LTR: Long terminal repeats
MITEs: Miniature inverted terminal repeats
MPC: Months post-conception
NHEJ: Non-homolog end joining
PTM: Post translational modifications
TE: Transposable element
TIR: Terminal inverted repeats
ZNF: Zing finger
