# Supplementary figures and images for: SETMAR, a case of primate co-opted genes: towards new perspectives
Source: Mob DNA. 2022 Apr 8;13:9. doi: 10.1186/s13100-022-00267-1 (PMC8994322; doi:10.1186/s13100-022-00267-1)

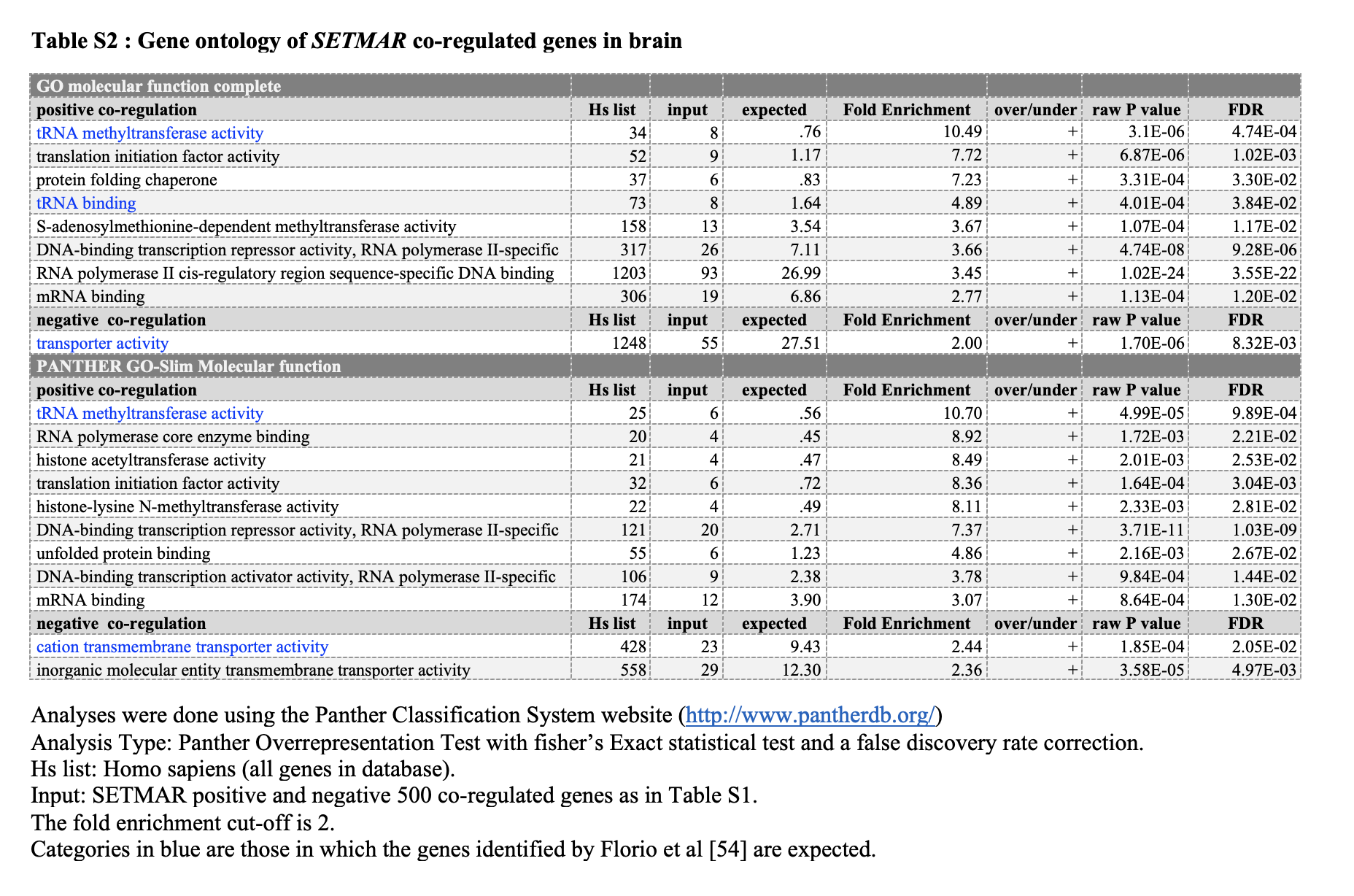

Supplement: Supplementary file 2 — Additional file 2: Table S2. Gene ontology of SETMAR and brain co-regulated genes during embryogenesis. [file 13100_2022_267_MOESM2_ESM.pdf]
