# Supplementary material for: SETMAR, a case of primate co-opted genes: towards new perspectives
Source: Mob DNA. 2022 Apr 8;13:9. doi: 10.1186/s13100-022-00267-1 (PMC8994322; doi:10.1186/s13100-022-00267-1)
Supplement: Supplementary file 3 — Additional file 3: Supp Data. Mat & Met for SETMAR and cognition. [file 13100_2022_267_MOESM3_ESM.docx]

**Supplemental data**

**SETMAR, a case of primate co-opted genes: towards new perspectives.**

Oriane LIÉ, Sylvaine RENAULT, Corinne AUGÉ-GOUILLOU*

UMR 1253, iBrain, Université de Tours, Inserm, Tours, France

**SETMAR and cognition: Materials and method**

*SETMAR expression during human embryogenesis*.

SETMAR expression data during human brain development for each brain structure were obtained from Ensembl Gene expression atlas.

(<http://www.ensembl.org/Homo_sapiens/Gene/ExpressionAtlas?db=core;g=ENSG00000170364;r=3:4303304-4317567>). Prism software was used to produce the visualization of SETMAR expression in function of developmental stages (Main text, Fig 3b). The Prism Spearman (3C) test was then applicated to these results to detect if correlation (if any) could occur between SETMAR and developmental stages (Main text, Fig 3c).

*SETMAR networks in brain*.

RNA expression data from Developmental Transcriptome Brainspan database (<http://www.brainspan.org/rnaseq/search/index.html>) have been used to identify SETMAR co-expressed genes during human brain development. Looking for correlates in all structures and during embryo development (8pcw-38pcw) resulted in a list of 52,376 SETMAR co-expressed genes, either positively or negatively correlated. For both, the 500 most correlated genes, with Pearson correlation factor r from strong (0.5) to very strong (0.9), were retained for further analyses. The 500 most positively correlated genes have r factors ranging from 0.993 to 0.648 and the 500 most negatively correlated genes have r factors ranging from -0.557 to -0.834. Both list are presented in table S1.

Both list were used to perform GeneOntology analysis, using Panther software with standard analysis options (Fisher's Exact test with False Discovery Rate).

To build the Tables 3A and 3B of the main text, Venn diagram were produced using genes containing at least one *Hsmar1* TIR (Renault *et al*, ref 42 of the main text), the 500 positively or negatively correlated expressed genes previously listed, the 50 genes from Florio *et al* study (ref 56 of the main text) and the 816 ID genes listed at the IDGenetics web site (<http://www.ccgenomics.cn/IDGenetics/index.php>).
